# Association of *IL12B* polymorphisms with susceptibility to Graves ophthalmopathy in a Taiwan Chinese population

**DOI:** 10.1186/1423-0127-19-97

**Published:** 2012-11-19

**Authors:** Yu-Huei Liu, Ching-Chu Chen, Li-Ling Liao, Lei Wan, Chang-Hai Tsai, Fuu-Jen Tsai

**Affiliations:** 1 Department of Medical Genetics and Medical Research, China Medical University Hospital, Taichung, Taiwan; 2 Graduate Institute of Integrated Medicine, China Medical University, Taichung, Taiwan; 3 Division of Endocrinology and Metabolism, Department of Medicine, China Medical University Hospital, Taichung, Taiwan; 4 Department of Endocrinology and Metabolism, College of Chinese Medicine, China Medical University, Taichung, Taiwan; 5 Department of Health Management, I Shou University, Kaohsiung, Taiwan; 6 School of Chinese Medicine, China Medical University, Taichung, Taiwan; 7 Department of Pediatrics, China Medical University Hospital, Taichung, Taiwan; 8 Asia University, Taichung, Taiwan; 9 School of Post-Baccalaureate Chinese Medicine, China Medical University, Taichung, Taiwan; 10 Department of Biotechnology, Asia University, Taichung, Taiwan; 11 Department of Biotechnology and Bioinformatics, Asia University, Taichung, Taiwan

## Abstract

**Background:**

*Interleukin 12B* (*IL12B*) gene polymorphisms have been linked to several inflammatory diseases, but their role in the development of Graves ophthalmopathy (GO) in Graves disease (GD) patients is unclear. The purpose of this study was to investigate the disease association of *IL12B* single nucleotide polymorphisms (SNPs).

**Methods:**

A Taiwan Chinese population comprising 200 GD patients with GO and 271 GD patients without GO was genotyped using an allele-specific extension and ligation method. Hardy-Weinberg equilibrium was estimated using the chi-square test. Allele and genotype frequencies were compared between GD patients with and without GO using the chi-square test.

**Results:**

The genotype and allele frequencies of examined SNPs did not differ between GD patients with and without GO. Although the genotype distribution remained nonsignificant in the sex-stratified analyses, the frequency of the *T* allele at SNP rs1003199 was significantly higher in patients with GO in the male cohort (*P* = 6.00 × 10^-3^). In addition, haplotypes of *IL12B* may be used to predict the risk of GO (*P* = 1.70 × 10^-2^); however, we could not prove the statistical significance of analysis after applying the Bonferroni correction.

**Conclusions:**

Our results provide new information that the examined *IL12B* gene polymorphisms may be associated with susceptibility to GO in the Taiwan Chinese population in a sex-specific manner. This conclusion requires further investigation.

## Background

Graves ophthalmopathy (GO) is a type of fibroproliferative autoimmune disease, which is characterized by inflammation, remodeling, and expansion of the retroocular fibroblasts of the eye. This disease commonly occurs in patients with Graves disease (GD) and serves as a major characteristic that is observed in 25–50% of patients with GD [1-5]. Although GO may develop from the activity of thyroid-stimulating hormone (TSH) receptor-stimulating antibodies [6-9], which are strongly associated with hyperthyroidism and GO, the mechanisms linking GD and GO remain largely elusive [10-12]. Recently, studies have suggested that cytokines may play a crucial role in regulating the development of GD and GO [13]; however, the detailed mechanisms thereof remain largely unclear.

*Interleukin 12B* (*IL12B*; OMIM 161561), which is located on chromosomal region 5q33.3, encodes the p40 subunit of the cytokines IL12 and IL23, which play a pivotal role in both innate and adaptive immunity [14]. Although the role of interleukins in the development of GO in GD patients is little known, thyroid hormones have been known to increase the secretion of IL12, which regulates dendritic cell maturation and function [15]. IL12 has been reported to be overexpressed during the active phase of GD and GO [16,17]. On the other hand, IL23 expression could be induced in fibroblast-like synoviocytes through active IL1B, a well-known GO-related cytokine in rheumatoid arthritis, in AP-1–dependent and nuclear factor-kappa B–dependent pathways [18-21]. These studies implied that p40, which is encoded by *IL12B*, might be involved in regulating inflammation.

To date, several single nucleotide polymorphisms (SNPs) in *IL12B* have been associated with various immune dysregulations such as systemic lupus erythematosus [22], type 1 diabetes [23], psoriasis and psoriatic arthritis [24], and GD [25]. Although the SNPs and expression of *IL12B* may be associated with autoimmune thyroid diseases, especially GD and GO in Western male populations [26,27], a genetic association between *IL12B* and GO in oriental populations has not been identified [28]. The aim of this study was to investigate the frequency of *IL12B* variants among GD patients and correlate it with the development of GO in a Taiwan Chinese population.

## Methods

### Patients

A study population of 471 GD patients with or without GO from China Medical University Hospital in Taiwan were enrolled using the inclusion/exclusion criteria summarized in Table 1, which has been published elsewhere [18,29,30]. All the methods followed the tenets of the Declaration of Helsinki. All participants signed the consent form approved by the Institutional Review Board of the China Medical University Hospital. GO was measured by the Hertel exophthalmometer. Patients who satisfied the NOSPECS system recommended by the American Thyroid Association [31] were classified as the GO group: (1) normal upper eyelid position 1.5 mm below the superior limbus and normal lower eyelid position at the level of the inferior limbus in primary gaze and (2) proptosis defined as the anteroposterior protrusion of the globe >19 mm from the lateral orbital rim in either eye or any discrepancy in the degree of protrusion of both eyes by >1 mm [32]. Those who did not satisfy both criteria were classified as the non-GO group. All individuals classified as affected were interviewed and examined by experienced clinicians.

**Table 1:** Inclusion/exclusion criteria

**Criteria**	**Details**
Inclusion	Patients must understand risks and benefits of the protocol and be able to give informed consent.
	Patient is a self-reported non-aboriginal Taiwanese, and none of the parents and grand-parents has aboriginal background.
	Patients must satisfy the diagnostic criteria of Graves disease at the time of examination.
Exclusion	Patients typical clinical features of hyperthyroidism, diffuse enlargement of the thyroid gland, increased free thyroxine or triiodothyronine levels, suppressed thyroid stimulating hormone levels, positive thyrotrophin-receptor autoantibodies, and with or without antimicrosomal or antithyroglobulin antibodies.
	Patient is unable to understand or give informed consent.
	Patients who had pregnancy or had delivered a baby/babies within one year.

### SNP selection

*IL12B* SNP genotype information from the HapMap CHB + JPT population was downloaded in December 2008. HapMap genotypes were analyzed within Haploview, and tag SNPs were selected using the Tagger function by applying (i) 0.1 as a threshold minor allele frequency (MAF) for “tag SNPs” in the HapMap CHB + JPT population and (ii) ≥0.6 as the Illumina score as recommended by the manufacturer. Eight polymorphisms within the *IL12B* gene were selected accordingly, including SNPs rs2195940 (*C/T* at intron 5), rs2853696 (*A/G* at intron 5), rs2421047 (*A/G* at intron 4), rs2853694 (*A/C* at intron 3), rs2569254 (*C/T* at intron 1), rs1003199 (*C/T* at intron 1), rs7709212 (*C/T* at promoter), and rs6868898 (*C/T* at promoter).

### Genomic DNA extraction and genotyping

Blood samples were collected by venipuncture and were subsequently subjected to genomic DNA isolation. A genomic DNA kit (Qiagen) was used for genomic DNA extraction. The DNA concentration of each sample was determined before genotyping. All 8 SNPs in the *IL12B* gene were analyzed using an allele-specific extension and ligation assay (Illumina) following the manufacturer’s instructions.

### Statistical analysis

Sample size was determined according to the previous report [33]. We set alpha as 0.05, where 0 and 1 represent *C* and *T* respectively for alleles at rs1003199, therefore the mean of all 198 male patients was 0.28 and the SD was 0.49). Associations between each SNP and GO were assessed using the chi-square test. An exact *P* value (two-tailed) < 0.05 with Bonferroni adjustment (threshold *P* value is 0.05/n, where n is number of all independent tests at the same time) [34] was considered statistically significant. The allelic and genotypic frequencies were compared in GD patients with and without GO, and the odds ratios (ORs) per allele/genotype were estimated by applying unconditional logistic regression with a 95% confidence interval (CI). All of the above statistical analyses were performed using PASW Statistics 18.0 software. ORs per haplotype were calculated using Haldane’s modification of Woolf’s equation: OR = [(a + 1/2) × (d + 1/2)]/[(b + 1/2) × (c + 1/2)], where a, b, c, and d represent the affected number in the GO group, the affected number in the non-GO group, the non-affected number in the GO group, and the non-affected number in the non-GO group, respectively, to avoid problems when critical entries are zero [35]. Screening for linkage disequilibrium was performed using Haploview ver. 4.1 [36]. The best locus–locus interaction models were detected with an estimate testing accuracy of >50% consistency using the multifactor dimensionality reduction (MDR) 1.1.0 of the open-source MDR software package (Dartmouth Medical School, Hanover, NH). The interaction dendrogram was established according to a hierarchical clustering algorithm [37-40].

## Results

### Genotypic frequencies of the *IL12B* polymorphisms in GD patients

All the samples from 471 GD patients were genotyped for the 8 SNPs in the *IL12B* gene. All of the tested SNPs in this study were found to be in Hardy-Weinberg equilibrium (*P* > 0.05, Table 2). To identify the SNPs associated with clinical features of GD, including GO, goiter, nodular hyperplasia, pretibial myxedema, and vitiligo, the 8 SNPs within the *IL12B* gene were investigated. None of these clinical features were associated with any of the tested *IL12B* polymorphisms (Table 3 and data not shown).

**Table 2:** **Eight single nucleotide polymorphisms in the *IL12B *gene identified from 471 Graves disease patients with or without Graves ophthalmopathy**

**SNPs**	**Position in *IL12*B**	**Chromosome position**	**Alleles**	**HWpval**	**MAF**
rs2195940	Intron 5	158744352	*C*:*T*	0.88	0.04
rs2853696	Intron 5	158744660	*G*:*G*	1.00	0.00
rs2421047	Intron 4	158746307	*A:G*	0.53	0.48
rs2853694	Intron 3	158749088	*A*:*C*	0.44	0.31
rs2569254	Intron 1	158751249	*C*:*T*	1.00	0.16
rs1003199	Intron 1	158755566	*C*:*T*	0.56	0.37
rs7709212	Promoter	158764177	*C*:*T*	0.22	0.49
rs6868898	Promoter	158764420	*C:T*	0.54	0.37

SNP positions in the *IL12B* gene were numbered upstream (+) or downstream (−) from the *IL12B* transcription initiation site. Chromosome positions were referred to the sequence of NCBI database (built 37.1). SNP, single nucleotide polymorphism; Hwpval, Hardy-Weinberg equilibrium *P* value; MAF, minor allele frequency.

**Table 3:** Frequencies of genotypes and alleles of single nucleotide polymorphisms in the IL-12B gene in Graves disease patients with or without Graves ophthalmopathy

**SNPs**	**GD/non-GO**	**GD/GO**	***P*value**^**a**^
	**Number (%)**	**Number (%)**	
rs2195940			
* C/C*	247 (91.14)	185 (92.50)	0.60
* C/T*	24 (8.86)	15 (7.50)	
* T/T*	0 (0.00)	0 (0.00)	
* C*	518 (95.57)	385 (96.25)	0.61
* T*	24 (4.43)	15 (3.75)	
rs2853696			
* A/A*	0 (0.00)	0 (0.00)	--
* A/G*	0 (0.00)	0 (0.00)	
* G/G*	271 (100.00)	200 (100.00)	
* A*	0 (0.00)	0 (0.00)	--
* G*	542 (100.00)	400 (100.00)	
rs2421047			
* A/A*	56 (20.66)	46 (23.00)	0.62
* A/G*	145 (53.51)	98 (49.00)	
* G/G*	70 (25.83)	56 (28.00)	
* A*	257 (47.42)	190 (47.50)	0.98
* G*	285 (52.58)	210 (52.50)	
rs2853694			
* A/A*	126 (46.49)	92 (46.00)	0.99
* A/C*	121 (44.65)	90 (45.00)	
* C/C*	24 (8.86)	18 (9.00)	
* A*	373 (68.82)	274 (68.50)	0.92
* C*	169 (31.18)	126 (31.50)	
rs2569254			
* C/C*	189 (69.74)	140 (70.00)	0.85
* C/T*	76 (28.04)	54 (27.00)	
* T/T*	6 (2.21)	6 (3.00)	
* C*	454 (83.76)	334 (83.50)	0.91
* T*	88 (16.24)	66 (16.50)	
rs1003199			
* C/C*	114 (42.07)	76 (38.00)	0.58
* C/T*	121 (44.65)	92 (46.00)	
* T/T*	36 (13.28)	32 (16.00)	
* C*	349 (64.39)	244 (61.00)	0.29
* T*	193 (35.61)	156 (39.00)	
rs7709212			
* C/C*	61 (22.51)	56 (28.00)	0.16
* C/T*	154 (56.83)	96 (48.00)	
* T/T*	56 (20.66)	48 (24.00)	
* C*	276 (50.92)	208 (52.00)	0.74
* T*	266 (49.08)	192 (48.00)	
rs6868898			
* C/C*	42 (15.50)	25 (12.50)	0.56
* C/T*	123 (45.39)	89 (44.50)	
* T/T*	106 (39.11)	86 (43.00)	
* C*	276 (50.92)	208 (52.00)	0.74
* T*	266 (49.18)	192 (48.00)	

Abbreviations: GD, Graves disease; GO, Graves ophthalmopathy; CI, confidence interval.

^a^*P* values (two-tailed) were calculated using chi-square tests with 3*×*2 contingency tables (genotypes; 2 d.f.) or 2*×*2 contingency tables (alleles; 1 d.f.).

### The *IL12B* polymorphism rs1003199 is significantly associated with the development of GO in male GD patients

In our study, there were many more female patients than male patients (female:male, 3.8:1). Because sex ratio is one variable that can affect the results of case–control association studies, a sex-stratification analysis was performed. Results showed that although no significant difference between *IL12B* SNPs and GO in the female patients was observed, SNP rs1003199 was significantly associated with the development of GO in the male GD patients: GD patients with the *T* allele at rs1003199 showed a 1.27-fold increase in the risk of developing GO compared with GD patients carrying the *C* allele (*P* = 6.00 × 10^-3^; OR = 2.27, 95% CI = 1.26–4.10; Table 4). The sample size was large enough (198 individuals) to detect the polymorphism (α = 0.05, power = 0.88). In addition, GD patients with a *T/T* genotype at rs1003199 showed a 7.85-fold increase in the risk of developing GO compared with GD patients carrying the *C/C* genotype (*P* = 0.01; OR = 8.85, 95% CI = 1.68–46.69; Table 4). However, only the significance of the allele distribution at rs1003199 remained after applying the Bonferroni correction. These results indicate that male GD patients carrying the *T* allele might be at a higher risk for developing GO than patients carrying other alleles, suggesting the possible role of *IL12B* in the development of GO in the male GD patients.

**Table 4:** **Allele frequencies of single nucleotide polymorphism in *IL-12B *among male Graves disease patients with or without Graves ophthalmopathy**

**SNPs**	**GD/nonGO**	**GD/GO**	***P *Value**^**a**^	**Odds ratio (95% CI)**^**b**^
	**Number (%)**	**Number (%)**		
rs2195940				
* C/C*	43 (89.58)	49 (96.08)	0.21	
* C/T*	5 (10.42)	2 (3.92)		
* T/T*	0 (0.00)	0 (0.00)		
* C*	91 (94.79)	100 (98.00)	0.22	
* T*	5 (5.21)	2 (2.00)		
rs2853696				
* A/A*	0 (0.00)	0 (0.00)	--	
* A/G*	0 (0.00)	0 (0.00)		
* G/G*	48 (100.00)	51 (100.00)		
* A*	0 (0.00)	0 (0.00)	--	
* G*	96 (100.00)	102 (100.00)		
rs2421047				
* A/A*	9 (18.75)	6 (11.76)	0.18	
* A/G*	32 (66.67)	30 (58.82)		
* G/G*	7 (14.58)	15 (29.41)		
* A*	50 (52.08)	42 (41.18)	0.12	
* G*	46 (47.92)	60 (58.82)		
rs2853694				
* A/A*	26 (54.17)	21 (41.18)	0.25	
* A/C*	20 (41.67)	24 (47.06)		
* C/C*	2 (4.16)	6 (11.76)		
* A*	72 (75.00)	66 (64.71)	0.11	
* C*	24 (25.00)	36 (35.39)		
rs2569254				
* C/C*	32 (66.67)	33 (64.71)	0.38	
* C/T*	16 (33.33)	16 (31.37)		
* T/T*	0 (0.0)	2 (3.92)		
* C*	80 (83.33)	82 (80.39)	0.59	
* T*	16 (16.67)	20 (19.61)		
rs1003199				
* C/C*	23 (47.92)	13 (25.49)	0.01	1
* C/T*	23 (47.92)	28 (54.90)		4.11 (0.82-20.66)
* T/T*	2 (4.16)	10 (19.61)		8.85 (1.68-46.69)
* C*	69 (71.88)	54 (52.94)	6.00×10^-3^	1
* T*	27 (28.12)	48 (47.06)		2.27 (1.26-4.10)
rs7709212				
* C/C*	14 (29.17)	12 (23.53)	0.82	
* C/T*	27 (56.25)	31 (60.78)		
* T/T*	7 (14.58)	8 (15.69)		
* C*	55 (57.29)	55 (53.92)	0.63	
* T*	41 (42.71)	47 (46.08)		
rs6868898				
* C/C*	5 (10.42)	10 (19.61)	0.19	
* C/T*	18 (37.50)	23 (45.10)		
* T/T*	25 (52.08)	18 (35.29)		
* C*	28 (29.17)	43 (42.16)	0.06	
* T*	68 (70.83)	59 (57.84)		

Abbreviations: SNP, single nucleotide polymorphism; GD, Graves disease; GO, Graves ophthalmopathy; CI, confidence interval.

^a^*P* values (two-tailed) were calculated using chi-square tests with 3*×*2 contingency tables (genotypes; 2 d.f.) or 2*×*2 contingency tables (alleles; 1 d.f.).

^b^Odds ratios and 95% CI per genotype and allele were estimated by applying unconditional logistic regression. *P* values less than 0.05 were considered significant.

### Frequencies of the *IL12B* haplotypes and their associations with GD and GO

Because our MDR analysis indicated that the best interaction model for the prediction of GO development in male GD patients is the 3-locus model (balance accuracy, 61.40%; cross-validation consistency = 100/100, *P* = 1.20 × 10^-3^; Table 5), we further estimated the association between the haplotype comprising the 3 SNPs rs1003199, rs7709212, and rs6868898 and GO. These results are listed in Table 6. In the male GD patients, the frequencies of *IL12B* haplotypes were significantly different (*P* = 1.70 × 10^-2^). Although haplotype-specific analysis showed that haplotypes HA5-*TCC* and HA7-*TTC* are associated with a higher risk of GO (*P* = 0.03; OR: 4.13 and *P* = 0.02; OR: 10.89; respectively), and haplotype HA2-*CCT* is associated with a lower risk of developing GO (*P =* 0.03; OR: 0.51), we could not prove the statistical significance of the analysis after applying the Bonferroni correction.

**Table 5:** Summary of multifactor dimensionality reduction (MDR) models for gene-gene interaction in male Graves disease patients with Graves ophthalmopathy

**Number of factors**	**Best candidate model**	**Balance accuracy**	**CV consistency**	***X***^**2**^	***P *value**	**Odds ratio (95%CI)**
1	rs1003199	59.47%	100/100	7.53	6.00×10^-3^	2.27 (1.26-4.10)
2	rs2195940 rs1003199	59.50%	76/100	7.67	6.00×10^-3^	2.30 (1.27-4.17)
3	rs1003199 rs7709212 rs6868898	61.40%	100/100	10.52	1.20×10^-3^	2.59 (1.45-4.62)
4	rs2853694 rs1003199 rs7709212 rs6868898	53.28%	100/100	12.47	4.00×10^-4^	2.82 (1.58-5.06)

**Table 6:** Estimated frequencies of the haplotypes inferred from rs1003199, rs7709212 and rs6868898 among male Graves disease patients with or without Graves ophthalmopathy

**Haplotype**		** SNP**		**GD/nonGO**	** GD/GO**	***P *Value**^**a**^	***P *Value**^**b**^	**Odds ratio**^**c**^
	**rs1003199**	**rs7709212**	**rs6868898**	**Number (%)**	**Number (%)**			
HA1	*C*	*C*	*C*	17 (17.71)	22 (21.57)	1.70 × 10^-2^	0.50	1.27
HA2	*C*	*C*	*T*	34 (35.42)	22 (21.57)		0.03	0.51
HA3	*C*	*T*	*C*	8 (8.33)	4 (3.92)		0.19	0.48
HA4	*C*	*T*	*T*	10 (10.42)	6 (5.88)		0.24	0.55
HA5	*T*	*C*	*C*	0 (0.00)	5 (4.90)		0.03	10.89
HA6	*T*	*C*	*T*	4 (4.17)	6 (5.88)		0.58	1.38
HA7	*T*	*T*	*C*	3 (3.13)	12 (11.76)		0.02	3.69
HA8	*T*	*T*	*T*	20 (20.83)	25 (24.51)		0.54	1.23

Abbreviations: GD, Graves disease; GO, Graves ophthalmopathy; CI, confidence interval.

^a^*P* values (two-tailed) were calculated using chi-square tests with 8×2 contingency tables (haplotypes; 7 d.f.).

^b^*P* values (two-tailed) were calculated using chi-square tests with 2×2 contingency tables (haplotypes; 1 d.f.).

^c^Odds ratio was calculated using Haldane’s modification of Woolf’ equation.

## Discussion

Several reports have documented that genetic factors may play a role in the development of GO [12,13,41,42]: the susceptibility genes for GO may include those encoding the *major histocompatibility complex* (*HLA*) *class I* and *class II* molecules [12], *cytotoxic T-lymphocyte-associated protein 4* (*CTLA4*) [12], cell surface molecules *intercellular adhesion molecule 1* (*ICAM1*) [43], and *integrin alpha E* (*ITGAE*) [29], as well as a variety of immunomodulatory genes, including those encoding the cytokines *interferon gamma* (*IFNG*) [26,44], *tumor necrosis factor* (*TNF*) [26,45], and *interleukin 1 beta* (*IL1B*) [18-20]. *IL12B* has been identified to possibly influence the development of autoimmune thyroid diseases and the related ophthalmopathy in male patients in the Western world [26,27], but it has not yet been deeply examined in Oriental patients [28]. In this study, we investigated whether *IL12B* SNPs contributed to the development of GO in a Taiwan Chinese GD population. The results show that none of the tested *IL12B* SNPs were associated with any manifestations of GD. However, the *T* allele at SNP rs1003199 was significantly associated with the occurrence of GO in male patients. Our results, in part, support the hypothesis that SNPs of pro-inflammatory cytokines may be related to GO development.

Theoretical and practical studies suggested that MDR has reasonable power for detecting interactions. Compared to the conventional statistical approaches, MDR was a novel, nonparametric, and genetic model–free approach that was developed specifically to detect not only interactions within or among genes but also interactions between genes and the environment. The MDR method has been used in several diseases such as cancers and inherited diseases [46,47]. However, a disadvantage of this method is that it overemphasizes the large dissimilarities and does not appropriately represent the small dissimilarities. Here, we used the MDR method to identify the interaction between 3 SNPs, including rs1003199 in intron 1 and rs7709212 and rs6868898 in the promoter, and GO. In addition, our preliminary results from a database search (MiRbase, http://www.mirbase.org/search.shtml) suggest that these SNPs may be miRNA targets. The *C* allele at rs6868898 showed higher similarity with miRNA sequences than the *T* allele at rs6868898. Moreover, only the *T* allele at rs1003199 and the *T* allele at rs7709212, but not the *C* allele at rs1003199 and the *C* allele at rs7709212, showed a similarity with miRNA sequences (Additional file 1). Whether these SNPs, as well as haplotypes, play a role in the development of GO by regulating the expression of the *IL12B* gene or the stability of the mRNA by miRNA needs further investigation.

The SNP rs6887695, which is located in the promoter region of the *IL12B* gene, may be associated with GO [26,27]. Although it was not selected under our SNP criteria, we also tried to analyze its genotype distribution in our patients, but we failed to find a statistically significant association between SNP rs6887695 and GO. Ethnic differences may be a reason for this discrepancy. Although the results presented here provide new information that may facilitate the understanding of the pathogenesis of GO, additional studies are necessary to confirm this finding in a larger sample and ideally, in a second group of affected and unaffected individuals.

The results of this study also provide novel information for identifying the mechanisms underlying the pathogenesis of GO. It has long been established that GO is a complex disease with a variety of weak-effect genes that are influenced by a wide range of environmental factors such as sex, iodine status, smoking, infectious agents, and stress. However, the reasons for the significant female preponderance of GD and GO are not well understood, but they may include skewed X chromosome inactivation [48], fetal microchimerism [49], or sex hormone effects [50]. In view of the significant sex-specific variation in GO, genetic factors may influence the establishment of differential risk between affected women and men. This study demonstrated that the polymorphisms of the *IL12B* gene may influence the development of GO. However, because of the lack of a larger sample size and published functional studies on these polymorphisms, the mechanisms of the associated polymorphisms need to be fully examined for their capacity to alter the GO phenotype. In addition, to confirm the disease-associated SNPs, further investigations on the role of the *IL12B* gene in GO development at the molecular level are warranted.

## Conclusion

In summary, this study showed that the *IL12B* polymorphisms may be associated with GO in Taiwan Chinese GD patients in a sex-specific manner. Whether these results can provide valuable insights requires further genetic and biological studies of the specific SNPs and haplotypes.

## Abbreviations

GD: Graves disease; GO: Graves ophthalmopathy; SNP: Single nucleotide polymorphisms; IL12B: Interleukin 12B; MAF: Minor allele frequency; OR: Odds ratio; CI: Confidence interval; MDR: Multifactor dimensionality reduction; HLA: Major histocompatibility complex; CTLA4: Cytotoxic T-lymphocyte-associated protein 4; ICAM1: Cell surface molecules intracellular adhesion molecule 1; ITGAE: Integrin alpha E; IFNG: Interferon gamma; TNF: Tumor necrosis factor; IL1B: Interleukin 1 beta.

## Competing interests

The authors declare that they have no competing interests.

## Authors' contributions

YHL: conception, design, analysis, statistical and logistical support, interpretation, literature search and article writing of the study; CCC: provision of patients, data collection and critical review of the study; LLL: statistical and logistical support of the study; LW: technique support of the study; CHT: obtaining funding of the study; FJT: obtaining funding of the study and final approval of the article. All authors read and approved the final manuscript.

## Supplementary Material

Additional file 1 **Table S1.** Prediction of polymorphisms of biological relevance in miRNA target sites. Click here for file
