# Supplementary material for: Association of IL12B polymorphisms with susceptibility to Graves ophthalmopathy in a Taiwan Chinese population
Source: J Biomed Sci. 2012 Nov 19;19(1):97. doi: 10.1186/1423-0127-19-97 (PMC3514134; doi:10.1186/1423-0127-19-97)
Supplement: Additional file 1 — Table S1. Prediction of polymorphisms of biological relevance in miRNA target sites. [file 1423-0127-19-97-S1.docx]

**Table S1. Prediction of polymorphisms of biological relevance in miRNA target sites.**

| **Polymorphisms** | **Input sequence** | **Accession** | **ID** | **Strand** | **Score** | **Evalue** |
| --- | --- | --- | --- | --- | --- | --- |
| rs1003199_*C* | CTAGAGGAAGAGGTAAGGGAACAATT[C]GGTATCTGTGTTTTATATATCATGG | - | - | - | - | - |
| rs1003199_*T* | CTAGAGGAAGAGGTAAGGGAACAATT[T]GGTATCTGTGTTTTATATATCATGG | MIMAT0000100 | hsa-miR-29b-3p | + | 60 | 4.9 |
|  |  | MIMAT0021023 | hsa-miR-5002-5p | + | 57 | 8.8 |
| rs7709212_*C* | ATGGATGCATTTCACCAGGTCACTGC[C]GAAATGTTGTACTTTTATGGATGGT | - | - | - | - | - |
| rs7709212_*T* | ATGGATGCATTTCACCAGGTCACTGC[T]GAAATGTTGTACTTTTATGGATGGT | MIMAT0021020 | hsa-miR-5000-3p | + | 58 | 7.3 |
| rs6868898_*C* | TCTGCCCTAACTGTCCCCATCTTGCT[C]CTGGGTATCTTTGCTCATTCCTGGG | MIMAT0019217 | hsa-miR-3189-5p | + | 64 | 2.3 |
|  |  | MIMAT0000279 | hsa-miR-222-3p | + | 62 | 3.4 |
|  |  | MIMAT0005877 | hsa-miR-1286 | - | 57 | 8.8 |
| rs6868898_*T* | TCTGCCCTAACTGTCCCCATCTTGCT[T]CTGGGTATCTTTGCTCATTCCTGGG | MIMAT0000279 | hsa-miR-222-3p | + | 62 | 3.4 |
